# Chemical Composition, Cytotoxic and Antimicrobial Activity of Essential Oils from *Cassia bakeriana* Craib. against Aerobic and Anaerobic Oral Pathogens

**DOI:** 10.3390/molecules18044588

**Published:** 2013-04-18

**Authors:** Luís C. S. Cunha, Sérgio A. L. de Morais, Carlos H. G. Martins, Mário M. Martins, Roberto Chang, Francisco J. T. de Aquino, Alberto de Oliveira, Thaís da S. Moraes, Fabrício C. Machado, Cláudio V. da Silva, Evandro A. do Nascimento

**Affiliations:** 1 Laboratory of Natural Products and Chromatography, Chemistry Institute, Federal University of Uberlândia, Uberlândia, 38408-144, Brazil; 2 Nucleus of Research in Sciences and Technology, Laboratory of Research in Applied Microbiology (LaPeMA), University of Franca, Franca, 14404-600, Brazil; 3 Institute of Biomedical Sciences, Laboratory of Molecular Biology, Federal University of Uberlândia, Uberlândia, 38400-902, Brazil

**Keywords:** *Cassia bakeriana*, essential oil, antimicrobial activity, cytotoxicity

## Abstract

The chemical composition of the essential oils from leaves, bark and wood of *Cassia* *bakeriana* Craib. was determined by gas chromatography (GC) and gas chromatography–mass spectrometry (GC-MS). Alcohols, aldehydes and fatty acids were the major components in leaf and bark oil, while wood essential oil was rich in fatty acids. Terpenes such as linalool, (*E*)-nerolidol and phytol were present in low concentrations. The antimicrobial activity against aerobic and anaerobic oral bacteria was evaluated using the microdilution method, as was the cell viability test carried out with Vero cells. The oils from leaves and bark showed high antimicrobial activity, with minimum inhibitory concentrations between 62.5 and 125 µg·mL^−1^ for most of the tested bacteria, including *Streptococcusmutans*, the main etiological agent of dental caries. Leaves oil displayed the lowest cytotoxic effect (EC_50_ of 153 µg·mL^−1^), while wood oil exhibited the highest toxicity to Vero cells. *C.* *bakeriana* oils are thus a source of biologically active compounds against aerobic and anaerobic oral microorganisms. This study is the first report on the chemical composition, antimicrobial activity and cytotoxicity of *C.bakeriana*.

## 1. Introduction

*Cassia bakeriana* Craib. is a tree belonging to the genus *Cassia* and the Fabaceae-Leguminoseae family, native to Thailand. It is also known as pink cassia and can reach 12 to 15 metres in height. Its original climate is humid tropical, but it tolerates well the subtropical conditions with mild winters of Brazil’s south and southeast regions [1]. This plant grows rapidly and shows good adaptation to the Brazilian cerrado. Many species of *Cassia* are used in folk medicine for their different pharmacological properties, such as laxative, purgative, antibacterial, antifungal and antimalarial activities, demonstrating the pharmacological potential of this plant genus. Studies of various parts of these species have reported the isolation of bioactive secondary metabolites, with anthraquinones, flavonoids and other phenolic compounds being the most common constituents present [2]. *C. bakeriana* leaves are considered an alternative source of laxative drugs and they exhibit a significant glycosylated anthraquinone content [3]. The chemical composition of essential oils present in plants has been studied, and these oils are promising sources of secondary metabolites with important biological properties, including antimicrobial activity [4]. A recent review revealed that several essential oils possess strong antimicrobial activity against various microorganisms, suggesting the possibility of using these oils as replacements of synthetic drugs to overcome the increasing resistance of some pathogens [5]. Some works have shown that essential oils are potential antibacterial agents against oral microorganisms [6,7,8,9]. These activities are generally related to secondary metabolite substances present in the oils. Several of these compounds were tested separately and have shown significant antimicrobial effects [10]. The chemical composition of the leaves oils of some species of *Cassia* such as *Cassia* *alata* [11,12] and *Cassia fistula* [13] has been determined.

The microorganisms evaluated in this study are considered to be risk factors for oral diseases such as caries and periodontitis; they can also reach the bloodstream triggering other diseases in the human body, like endocarditis, brain abscesses, throat infections, respiratory and gastrointestinal system infections and bacteremia. The oral cavity is one of the most complex microbiome in the human body. More than 700 bacterial species have been detected in this group by molecular methods [14]. The search for new natural products or prototypes with antibacterial activity would be very important for the control and prevention of oral and systemic diseases in human health. Given this perspective, the aim of the present study was to determine the chemical composition of the essential oils from leaves, bark and wood of *C. bakeriana*, and investigate their antimicrobial potential against aerobic and anaerobic oral pathogens and their cytotoxic effects. This is the first report involving the chemical composition and biological activity of essential oils of *C.* *bakeriana*.

## 2. Results and Discussion

### 2.1. Chemical Composition of the Essential Oils

Table 1 shows the composition of essential oils from the leaves, barks and wood of *C. bakeriana*. Table 2 shows the classification of compounds in the essential oil from different parts of *C. bakeriana* classified by functional groups. The leaves had the highest essential oil yield of 0.94 ± 0.03%, followed by barks at 0.46 ± 0.08%, and wood at 0.12 ± 0.02%. Surprisingly, terpenoids are represented only by linalool (3.55%) and phytol (4.45%) in the leaves’ essential oil.

**Table 1:** Chemical composition of essential oil from different parts of *C. bakeriana.*

Compound	AIReference	AI(Calculated)	Identificationmethod	Composition (%)		
				Wood	Bark	Leaves
Hexanal	801	806	a, b, c	1.13	0.82	-
Furfural	828	830	a, b, c	1.27	-	-
(*E*)-Hex-2-enal	846	847	a, b, c	-	-	10.48
(*Z*)-Hex-3-en-1-ol	850	851	a, b, c	1.56	6.73	34.90
Hex-2-en-1-ol	854	855	a, b, c	-	-	0.95
Hexan-1-ol	863	862	a, b, c	1.03	0.72	-
Octanal	998	1001	a, b, c	-	0.87	-
Hex-3-en-1-ol, acetate	1004	1007	a, b, c	-	-	8.68
Phenylacetaldehyde	1036	1038	a, b, c	3.76	-	-
Octan-1-ol	1063	1065	a, b, c	-	2.82	-
Linalool	1097	1097	a, b, c	-	-	3.55
Nonanal	1100	1099	a, b, c	-	14.42	-
Nonanol	1165	1170	a, b, c		0.98	
Methyl salicylate	1190	1193	a, b, c	-	1.27	-
Octanoic acid (caprylic acid)	1192	1196	a, c, d	-	1.53	-
Decanal	1201	1201	a, b, c	-	0.89	-
*Cis*-dec-2-enal	1261	1262	a, b, c	-	6.17	-
Nonanoic acid (pelargonic acid)	1293	1291	a, c, d	-	5.77	-
4-Vinylguaiacol	1309	1314	a, b, c	-	-	2.41
4-propylguaiacol	1374	1367	a, b, d	3.92	-	-
(*E*)-Nerolidol	1561	1564	a, b, c	-	4.70	-
Dodecanoic acid (lauric acid)	1565	1566	a, c, d	-	1.92	0.93
Tridecanoic acid (tridecylic acid)	1662	1660	a, c, d	-	0.79	-
Tetradecanoic acid (myristic acid)	1770	1764	a, c, d	-	1.45	-
N. I.	-	-	-	0.93	-	-
Hexadecanoic acid (palmitic acid)	1959	1958	a, c, d	58.14	34.80	5.89
N. I.	-	-	-	1.05	-	-
Phytol	2114	2110	a, c, d	-	-	4.45
(*Z*,*Z*)-Octadeca-9,12-dienoic acid (linoleic acid)	2132	2149	a, c, d	8.46	2.09	-
(*Z*)-Octadec-9-enoic acid (oleic acid)	2132	2149	a, c, d	15.22	2.20	-
Octadecanoic acid (stearic acid)	2158	2155	a, c, d	3.53	0.72	-
Tricosane	2300	2300	a, b, c	-	0.69	1.03
Tetracosane	2400	2400	a, b, c	-	-	1.14
N. I.	-	-	-	-	-	2.38
Pentacosane	2500	2500	a, b, c	-	1.33	-
Heptacosane	2700	2700	a, b, c	-	-	8.27
N. I.	-	-	-	-	3.62	-
Octacosane	2800	2800	a, b, c	-	0.74	2.07
N. I. (hydrocarbon)	-	-	-	-	2.06	12.57
N. I. (hydrocarbon)	-	-	-	-	0.93	-
Total identified (%)				98.02	93.39	86.05

AI = Arithmetic index on a DB5 column (comparison with n-alkanes C_8_-C_40_); N. I. = Not Identified. Identification method: (a) Arithmetic index; (b) Adams mass spectral-retention index library; (c) Mass spectrum comparison with the *WileyLibrary*; (d) mass spectrum and arithmetic index comparison with NIST Standard Reference Data (*NIST 2011*).

**Table 2:** Classification of compounds in the essential oil from different parts of *C. bakeriana* by functional groups (N.I. = not identified).

Functional groups	Wood	Bark	Leaves
Alcohols	2.59	11.15	37.15
Aldehydes	6.16	23.17	10.48
Esters	-	-	8.68
Oxygenated monoterpenes	-	-	3.55
Oxygenated sesquiterpenes	-	4.70	-
Oxygenated diterpenes	-	-	4.45
Long chain alkanes	-	2.76	12.78
Phenolics	3.92	-	2.41
Fatty acids	85.35	51.27	6.82
N. I. compounds	1.98	6.61	13.95

**Figure 1:**
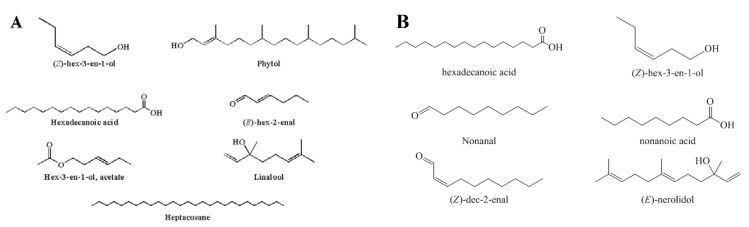
Major componentsin the leaf oil (**A**) and bark (**B**) of *C. bakeriana.*

The major components identified in the leaves were (*Z*)-hex-3-en-1-ol (34.90%), heptacosane (8.27%), hex-3-en-1-ol, acetate (8.6%), (*E*)-hex-2-enal (10.48%), hexadecanoic acid (5.89%), the oxygenated diterpene phytol (4.45%) and the oxygenated sesquiterpene linalool (3.55%) (Figure 1). Most of these compounds differ significantly from those found in other species of *Cassia*. In previous studies of essential oil of *C. alata* leaves, the major compounds were linalool (23.0%), borneol (8.6%) and pentadecanal (9.3%) [11], and 1,8-cineole (39.8%), β-caryophyllene (19.1%) and caryophyllene oxide (12.7%) [12]. Leaves of *C. fistula* contained as the main constituents phytol (16.1%), tetradecane (10.5%) and hexadecane (8.7%) [13]. The chemical composition of essential oils can vary among species, among the same species and the different plant parts. These differences are due to several factors that affect the oil chemical composition such as climate, soil quality, harvest season and genetics. In general, at low intensity of light the production of monoterpenes decreases and a small daily variation of temperature can stimulate the production of terpenoids [15]. 

The wood oil contained fatty acids as the major components, which accounted for 85.35% of the total, including palmitic (58.14%), oleic (15.22%) and linoleic (8.46%) acids as the major ones. Phenylacetaldehyde (3.76%) and 4-propylguaiacol (3.92%) were also present in significant amounts. 

The bark oil was rich in hexadecanoic acid (34.80%), nonanal (14.42%), (*Z*)-hex-3-en-1-ol (6.73%), (*Z*)-dec-2-enal (6.17%), nonanoic acid (5.77%) and in the oxygenated sesquiterpene (*E*)-nerolidol (4.70%) (Figure 1). Fatty acids were also the predominant class of compounds in the essential oil of bark at 51.27%.

### 2.2. Antimicrobial Activity of Essential Oils

The antimicrobial activity of different plant parts against aerobic and anaerobic oral microorganisms was determined, as was their cytotoxicity. The antimicrobial activity and cytotoxicity results are shown in Table 3. The leaves and bark oils indicated strong antibacterial activity, showing inhibition of both types of microorganisms. The MIC values ranged from 62.5 to 125 µg·mL^−1^ for most of the bacteria tested. All bacteria demonstrated some degree of sensitivity to the essential oils within the concentrations tested. The more susceptible microorganisms were *A. naeslundii*, *P. gingivalis* and *B. fragilis* from anaerobic bacteria and *S. mitis*, *S. mutans*, *S. sanguinis* and *A. actinomycetemcomitans* from aerobic bacteria, while *F. nucleatum* was more resistant. The wood essential oil exhibited lower activity against investigated bacteria with MICs ranging from 500 to 2000 (or higher) µg·mL^−1^. 

**Table 3:** Inhibitory effect of essential oils against aerobic and anaerobic oral bacteria and cytotoxic activity of different parts of *C. bakeriana*.

Microorganisms		Minimum inhibitory concentration (MIC) – μg·mL^−1^			
		Samples of Essential Oils			
		Leaves	Bark	Wood	^a^ CHD
Anaerobic	^c^ *F. nucleatum* (ATCC 25586)	1000	1000	2000	0.922
	^b^ *A. naeslundii* (ATCC 19039)	62.5	125	1000	1.844
	^c^ *P. gingivalis*(ATCC 48417)	125	125	500	3.68
	^c^ *B. fragilis* (ATCC 25285)	62.5	62.5	1000	1.844
Aerobic	^b^ *S. sanguinis* (ATCC 10556)	125	125	1000	3.68
	^b^ *S. mitis* (ATCC 49456)	62.5	62.5	500	3.68
	^b^ *S. mutans* (ATCC 25175)	62.5	62.5	2000	0.922
	^c^ *A. actinomycetemcomitans* (ATCC 43717)	125	125	1000	14.7
Cytotoxic activity EC_50_ – μg·mL^−1^ Vero cells (ATCC CCL 81)		153 ± 13	119 ± 2	93 ± 3	----

^a^ CHD: positive control (chlorhexidine dihydrochloride); ^b^ Gram-positive bacteria; ^c^ Gram-negative bacteria.

Comparing Table 1, Table 3, the similar results presented by leaf and bark essential oils could be assigned to the identified aliphatic alcohols and aldehydes. On the other hand, fatty acids, which were present in high concentrations in the wood essential oil, apparently had no inhibitory effects on the tested bacteria. Usually, the biological properties of essential oils are determined by the characteristics of the major components [16]. The antimicrobial activity in this study may be related to a specific metabolite that is in highest concentration in the oil composition or a synergism between major and minor compounds in the mixture. Unidentified compounds in oils can be contributors to the antimicrobial effects by reinforcement or synergistic interactions. Some compounds found in the essential oils are well-known antimicrobial agents, such as (*Z*)-hex-3-en-1-ol, linalool [17], caprylic acid [18], lauric and linoleic acids [19], aldehydes, particularly (*E*)-hex-2-enal [20], methyl salicylate [21], (*E*)-nerolidol [22] and octan-1-ol [23].

Although they did not have a high composition of terpenes, the essential oils of the leaves and bark of *C. bakeriana* showed significant antimicrobial activity against the oral pathogens evaluated. Extracts or oils from plant species with MIC values below 100 µg·mL^−1^ are considered promising as potential antimicrobial agents [24]. The essential oils of the leaves and barks of *C. bakeriana* exhibited strong antibacterial effect against oral microorganisms showing MIC values lower than 100 µg·mL^−1 ^ and lower than other studies in the literature. The leaf oil of *Lippia sidoides* and pure standards of monoterpenes (thymol and carvacrol) against *S. mutans*, *S. mitis*, *S. sanguinis* and *S. salivarus,* showed MICs from 2,500 µg·mL^−1^ to 10,000 µg·mL^−1^ [7]. The MIC values of the essential oil of *C. pubescens* for the same microorganisms studied in this work ranged from 500 µg·mL^−1^ to 2000 µg·mL^−1^ [9]. Oils of *Leptospermum scoparium*, *Melaleuca alternifoia*, *Eucalyptus radiata* e *Lavandula officialis* inhibited the growth of *S. mutans*, *S. sobrinus*, *A. actinomycetemcomitans*, *P. gingivalis* and *F. nucleatum* with MICs ranging from 300 µg·mL^−1^ to 10,000 µg·mL^−1^ [6] The essential oil of *Artemisia iwayomogi* was evaluated against fifteen oral bacteria, and inhibitions occurred with values between 100 µg·mL^−1^ to 3,200 µg·mL^−1^, except for *P. gingivalis* with an MIC of 50 µg·mL^−1^ [8]. Using a concentration of 62.5 µg·mL^−1^, leaf and bark oils of *C. bakeriana* inhibited the growth of *S. mutans*, principal etiological agent of dental caries. The results are even more promising because both oils exhibited higher toxicity to the microrganisms than to Vero cells at the same concentration. A comparison of the cytotoxicity to Vero cells and antimicrobial activity was performed using the selectivity index, which was calculated by the logarithm of the ratio of cytotoxic concentration (EC_50_) and the MIC value for microorganisms (SI = log [EC_50_]/[MIC]). A positive value represents higher selectivity against microorganisms than toxicity to Vero cells, and a negative value indicates a higher toxicity to Vero cells than to bacteria [25]. The selectivity index for the leaf oil at inhibitory concentrations of 62.5 µg·mL^−1^ and 125 µg·mL^−1^ were 0.38 and 0.08 respectively and for the bark oil in the same concentrations were 0.28 and −0.021 respectively. For the oil wood the obtained SI indexes were −0.73 and −1.03 at the concentrations of 500 and 1000 µg·mL^−1^ respectively, indicating greater toxicity to Vero cells and low activity against oral pathogens. According to the MIC values and selectivity indexes, the leaf oil showed promising antibacterial activity while simultaneously displaying low cytotoxic effects to Vero cells.

## 3. Experimental

### 3.1. Plant Material

Different parts of *C. bakeriana* (leaf, bark and wood) were collected in June 2011 at the Federal University of Uberlândia, Minas Gerais, Brazil (18°55'8.95"S; 48°15'34.01"W). The plant is located in the Cerrado area (savannah). The plant was identified by a specialist, and a voucher specimen was deposited in the Uberlandenses Herbarium, under number 63584, in Uberlândia.

### 3.2. Essential Oil Isolation

Flesh leaves, barks and wood of *C. bakeriana* were separately cut and about 400 grams of each part were extracted for four hours by hydrodistillation using a Clevenger-type apparatus. The oil obtained was extracted with dichloromethane (5.0 mL), the organic layer was separated and this fraction was dried with anhydrous sodium sulfate, filtered and kept in a closed vial under refrigeration (−10 °C) for further analysis. The percent yield was calculated relative to the dried mass of the initial sample.

### 3.3. Analysis of the Essential Oils

The oil was analysed by a Shimadzu equipment (Kyoto, Japan) model CG-17A/QP-5000 gas chromatograph coupled to a mass spectrometer (GC-MS), equipped with a 30-m DB-5 capillary column. The carrier gas used was helium at a flow rate of 1 mL/min, detector and injector temperatures were 220 °C and 240 °C, respectively, and the injection volume was 1 µL and the split ratio was 50:1. The oven temperature was programmed from 60 to 240 °C at 3 °C/min. The electron impact energy was set at 70 eV and fragments from 40 to 650 *m/z* were collected. The quantification of oils were performed in a Shimadzu brand model 2014 GC/flame ionization detector (GC/FID) under the same conditions employed in the GC/MS, using N_2_/Air/H_2_ as burning and carrier gas. 

### 3.4. Identification of the Constituents

Identification of each individual constituent of the essential oils was achieved based on Arithmetic Index on DB-5 in reference do standard compounds; and by comparison of their mass spectral fragmentation patterns (NIST database/ChemStation data system and Wiley library/ChemStation data system mass spectral database) [26,27]. For calculation of arithmetic indexes for linear C_8_-C_40_ alkanes were calculate by equation based on alkane standards: AI (x) = 100 P_z_ + 100[(t (x) − t (P_z_))/t (P_z+1_) − t (P_z_))]; where x: compound at time t; P_z_: alkane before x; and P_z+1_: alkane after x [26].

### 3.5. Microbial Strains

The tested strains were obtained from the American Type Culture Collection (ATCC, Rockville MD, USA). The following microorganisms were used in the present work: aerobic *Streptococcus mutans* (ATCC 25175), *Streptococcus mitis* (ATCC 49456), *Streptococcus sanguinis* (ATCC 10556) and *Agregatibacter actinomycetemcomitans* (ATCC 43717) and anaerobic *Fusobacterium nucleatum* (ATCC 25586), *Bacteroides fragilis* (ATCC 25285), *Actonomyces naeslundii* (ATCC 19039) and *Porphyromonas gingivalis* (ATCC 48417).

### 3.6. Antimicrobial Activity

The MIC (minimal inhibitory concentration) value is the lowest concentration of the compound, extract or oil capable of inhibiting microorganism growth. The essential oils of different parts of *C. bakeriana* were determined in triplicate by using the microdilution broth method in 96-well microplates [28]. Samples were dissolved in dimethyl sulfoxide (DMSO; Synth, São Paulo, Brazil) at 8,000 µg·mL^−1^, followed by dilution in tryptic soy broth (Difco, Detroit, MI, USA) for aerobic and Schaedler broth (Difco) supplemented with hemin (5.0 μg·mL^−1^) and vitamin K1 (10.0 μg·mL^−1^) for anaerobic; concentrations tested ranged from 2000 to 10 µg·mL^−1^. The final DMSO content was 4% (v/v), and this solution was used as a negative control. The inoculum was adjusted for each organism, to yield a cell concentration of 5 × 10^5^ colony forming units (CFU) per mL, according to National Committee for Clinical Laboratory Standard (NCCLS) guidelines [29]. The microplates (96 wells) with the aerobic microorganisms were closed with a sterile plate sealer and incubated aerobically at 37 °C for 24 h. The anaerobic microorganisms were closed with a sterile plate sealer and incubated for 48–72 h in an anaerobic chamber (Don Whitley Scientific, Bradford, UK), in 5%–10% H_2_, 10% CO_2_, 80%–85% N_2_ atmosphere at 37 °C. After that, resazurin (30 µL) in aqueous solution (0.01%) was added to the microplates, to indicate microorganism viability for the determination of minimal inhibitory concentration [28]. Chlorhexidine dihydrochloride (CHD) was used as a positive control, and the concentrations ranged from 0.0115 µg·mL^−1^ to 5.9 µg·mL^−1^. The controls of sterility of TSB and Schaedler broths, control culture (inoculum), chlorhexidine dihydrochloride sterility, sterility of the extracts and control DMSO were performed.

### 3.7. Cytotoxic Activity

Samples of the essential oils were dissolved in methanol and diluted in culture medium DMEM supplemented until form a solution with a concentration of 640 μg·mL^−1^. The cell viability test was performed with Vero ATCC CCL 81 cells (kidney fibroblasts African green monkey). For evaluation of cytotoxicity was used microplate dilution method. A solution containing 1 × 10^6^ cells in 10 µL supplemented DMEM was prepared, and 100 µL of this solution was pipetted into each well and then the plate was incubated for 6 h at 37 °C with humidified atmosphere and 5% CO_2_, occurring cell adhesion in the well. Once attached, the culture medium was removed and added solutions of the samples at concentrations of 512, 256, 128, 64, 32, 16, 8 and 4 µg·mL^−1^, starting from the initial solution of DMEM. The final volume in each well was 100 µL and the concentration of cells present in each well was 1.10^4^ cells. The final concentration of methanol in each well did not exceed 3%. Controls growth, negative (100% lysed cells), solvent (methanol) and samples were also prepared. The plates were incubated for 48 h at 37 °C with humidified atmosphere and 5% CO_2_. After, 10 uL of developing solution of 3 mM resazurin in PBS was added to each well [30] and the plate was incubated again for 24 h under the same conditions. Readings of absorbance at 590 nm was performed in a microplate spectrophotometer. The assays were carried out in triplicate and the results of the absorbances for each concentration tested were calculated according to the growth control. The EC_50_ (concentration at which 50% of the cells are viable) was calculated by a dose-response graph nonlinear regression [31].

### 3.8. Statistical Analysis

All data on the biological tests were submitted to treatment ANOVA with a significance level of 5%, using the Tukey method in GraphPad Prism 5.

## 4. Conclusions

The essential oils of *C. bakeriana* leaves and barks have shown promising activity against oral pathogens, including aerobic and anaerobic bacteria. Saturated and unsaturated aliphatic alcohols and aldehydes and terpenes or synergistic interactions between the major and minor constituents within the oils could be responsible for the observed inhibitory effects. The oil leaves indicated higher selectivity against oral pathogens than toxicity to Vero cells in the concentrations that inhibited the growth of most microorganisms evaluated. Our results indicated that some active components are present in oils, so this makes them particularly interesting for future studies and development of novel antimicrobial agents.

## References

[1] Lorenzi H., Souza H.M., Torres M.A.V., Bacher L.B. (2003). Árvores Exóticas no Brasil - madeireiras, *ornamentais e exóticas*.

[2] Júnior C.V., Rezende A., Silva D.H.S., Castro-Gambôa I., Bolzani V.S. (2006). Aspectos químicos, biológicos e etnofarmacológicos do gênero Cassia. Quim. Nova.

[3] Gritsanapan W., Phadungrakwitya R., Nualkaew S. Investigation of Alternative Anthraquinone Sources from Cassia spp.. Proceedings of International Conference on Development of Botanicals.

[4] Xiong L., Peng C., Zhou Q.M., Wan F., Xie X.F., Guo L., Li X.H., He C.J., Dai O. (2013). Chemical composition and antibacterial activity of assential oils from different parts of *Leonurus japonicus* Houtt. Molecules.

[5] Lang G., Buchbauer G. (2012). A review on recent research results (2008–2010) on essential oils as antimicrobials and antifungals. A review. Flav. Frag. J..

[6] Takarada K., Kimizuka R., Takarashi R., Honma K., Okuda K., Kato T. (2004). A comparison of the antibacterial efficacies of essential oils against oral pathogens. Oral Microbiol. Immunol..

[7] Botelho M.A., Nogueira N.A., Bastos G.M., Fonseca S.G., Lemos T.L., Matos F.J., Montenegro D., Heukelbach J., Rao V.S., Brito G.A. (2007). Antimicrobial activity of the essential oil from *Lippia sidoides*, carvacrol and thymol against oral pathogens. Braz. J. Med. Biol. Res..

[8] Cha J.D. (2007). Chemical composition and antibacterial activity against oral bacteria by the essential oil of *Artemisia iwayomogi*. J. Bacteriol. Virol..

[9] Chang R., Morais S.A.L., Nascimento E.A., Cunha L.C.S., Rocha E.O., Aquino F.J.T., Souza M.G.M., Cunha W.R., Martins C.H.G. (2011). Essential oil composition and antioxidant and antimicrobial properties of *Campomanesia pubescens* O. Berg, Native of Brazilian Cerrado. Lat. Am. J. Pharm..

[10] Si W., Gong J., Tsao R., Zhou T., Yu H., Poppe C., Johnson R., Du Z. (2006). Antimicrobial activity of essential oils and structurally related synthetic food additives towards selected pathogenic and beneficial gut bacteria. J. Appl. Microb..

[11] Agnaniet H., Bikanga R., Bessière J.M., Menut C. (2005). Essential oil constituents of *Cassia alata* (L.) from Gabon. J. Essent. Oil Res..

[12] Ogunwande I.A., Flamini G., Cioni P.L., Omikorede O., Azeez R.A., Ayodele A.A., Kamil Y.O. (2010). Aromatic plants growing in Nigeria: Essential oil constituents of *Cassia alata* (Linn.) Roxb. and *Helianthus annuus* L.. Rec. Nat. Prod..

[13] Tzakou O., Loukis A., Said A. (2007). Essential oil from the flowers and leaves of *Cassia fistula* L.. J. Essent. Oil Res..

[14] Aas J.A., Paster J.B., Stokes N.L., Olsen I., Dewhirst E.F. (2005). Defining the normal bacterial flora of the oral cavity. J. Clin. Microbiol..

[15] Lima R.K. (2008). Essential Oils of *Myristica fragrans* Houtt. and of *Salvia microphylla* H.B.K.: Chemical Characterization, Biological And Antioxidant Activity. Ph.D. Thesis.

[16] Bakkali F., Averbeck S., Averbeck D., Idaomar M. (2008). Biological effects of essential oils—A review. Food Chem. Toxicol..

[17] Dorman H.J.D., Deans S.G. (2000). Antimicrobial agents from plants: Antibacterial activity of plant volatile oils. J. Appl. Microbiol..

[18] Kollanoor A., Vasudevan P., Nair M.K.M., Hoagland T., Venkitanarayanan K. (2007). Inactivation of bacterial fish pathogens by medium-chain lipid molecules (caprylic acid, monocaprylin and sodium caprylate). Aquaculture Res..

[19] Ouattara B., Simard R.E., Holley R.A., Piette G.J.P., Bégin A. (1997). Antibacterial activity of selected fatty acids and essential oils against six meat spoilage organisms. Int. J. Food Microbial..

[20] Patrignani F., Iucci L., Belletti N., Gardini F., Guerzoni M.E., Lanciotti R. (2008). Effects of sub-lethal concentrations of hexanal and 2-(*E*)-hexenal on membrane fatty acid composition and volatile compounds of *Listeria monocytogenes*, *Staphylococcus aureus*, *Salmonella enteritidis* and *Escherichia coli*. Int. J. Food Microbial..

[21] Chung J.Y., Choo J.H., Lee M.H., Hwang J.H. (2006). Anticariogenic activity of macelignan isolated from *Myristica fragrans* (nutmeg) against *Streptococcus mutans*. Phytomedicine.

[22] Skaltsa H.D., Lazzari D.M., Mavromati A.S., Tiligada E.A., Constantinidis T.A. (2000). Composition and antimicrobial activity of the essential oil of *Scutellaria albida* ssp. albida from Greece. Planta Med..

[23] Togashi N., Shiraishi A., Nishizaka M., Matsuoka K., Endo K., Hamashima H., Inoue Y. (2007). Antibacterial activity of long-chain fatty alcohols against *Staphylococcus aureus*. Molecules.

[24] Rios J.L., Recio M.C. (2005). Medicinal plants and antimicrobial activity. J. Ethnopharmacol..

[25] Case R.J., Franzblau S.G., Wang Y., Cho S.H., Soejarto D.D., Pauli G.F. (2006). Ethnopharmacological evaluation of the informant consensus model on anti-tuberculosis claims among the Manus. J. Ethnopharmacol..

[26] Adams R.P. (2007). Identification of Essential Oil Components by Gas Chromatography/Quadrupole Mass Spectroscopy.

[27] NIST Standard Reference Data.

[28] Carvalho T.C., Simão M.R., Ambrósio S.R., Furtado N.A., Veneziani R.C., Heleno V.C., da Costa F.B., Gomes B.P., Souza M.G., Reis R.B. (2011). Antimicrobial activity of diterpenes from *Viguiera arenaria* against endodontic bacteria. Molecules.

[29] National Committee for Clinical Laboratory Standards (NCCLS) (2003). NCCLS document M7-A6 - Methods for Dilution Antimicrobial Susceptibility Tests for Bacteria That Grow Aerobically.

[30] Gómez-Barrio A., Veja C., Escario J.A., Rolón M. (2006). Development of resazurin microtiter assay for drug sensibility testing of *Trypanosoma cruzi* epimastigotes. Parasitology Res..

[31] Chibale K., Chouteau F., Lategan C.A., Maharaj V.J., Pillay P., Smith P.J., Vleggaar R. (2007). Antiplasmodial hirsutinolides from *Vernonia staehelinoides* and their utilization towards a simplified pharmacophore. Phytochemistry.

